# How individuals with psychosis develop and maintain resilience to suicidal experiences through psychological therapy: a qualitative study

**DOI:** 10.1186/s12888-024-06071-w

**Published:** 2024-12-03

**Authors:** N. Berry, S. Peters, G. Haddock, A. Scott, K. Harris, L. Cook, Y. Awenat, P. A. Gooding

**Affiliations:** 1 Division of Psychology and Mental Health, School of Health Sciences, Faculty of Biology, Medicine and Health, Manchester Academic Health Sciences Centre, University of Manchester, Coupland Building 1, Oxford Road, Manchester, M13 9PL UK; 2 Greater Manchester Mental Health NHS Foundation Trust, Manchester, UK

**Keywords:** Psychosis, Schizophrenia, Suicide, Resilience, Qualitative, Expert by experience and service user involvement

## Abstract

**Background:**

Almost half of people with psychosis have suicidal experiences. There is limited understanding of the processes underpinning psychological resilience to psychotic and suicidal experiences especially in people who have engaged with psychological talking therapies. Hence, the current study aimed to redress this gap by examining the perspectives of clients who had recent lived-experiences of psychosis, suicidality, and psychological therapy.

**Methods:**

Semi-structured interviews were conducted with 35 participants who had psychosis and suicidal experiences in the three months prior to recruitment. Data were analysed using reflexive thematic analysis.

**Results:**

There were four key psychological processes that contributed to resilience: (1) gaining a sense of control by nullifying perceptions of being controlled by overwhelming emotional and psychotic experiences, and instead, acquiring confident autonomy; (2) gaining a sense of hope facilitated by experiencing mental health problems on a fluctuating continuum, translating immersive positive memories into future possibilities, and embracing meaningful personal values; (3) developing genuine self-worth based on compassionate self-understanding, and affirmation of personal qualities; and (4) finding acceptance by being able to live alongside psychotic and suicidal experiences. Specific resilience components that participants developed through psychological therapy, together with their own knowledge and observations, were identified.

**Conclusions:**

People who live with psychotic and suicidal experiences can, and do, experience psychological change and psychological resilience in response to psychological therapy. To promote resilience, practitioners should pursue a person-based, client-directed approach; embrace acceptance; and be open to exploring different ways of working with clients’ experiences of overwhelming thoughts and emotions.

## Background

Suicidal experiences include thoughts, urges, compulsions, plans, and acts. Globally, over 700,000 people die by suicide every year [1]. In 2021, there were 5,583 registered deaths by suicide in England and Wales, which equates to 10.7 deaths per 100,000 people [2]. Individuals experiencing psychosis (e.g., hallucinations, delusions and/or paranoia) are at a higher risk of suicide than the general population [3] with almost half reporting current or past suicidal thoughts and/or behaviours [4]. Suicidal experiences in psychosis have been linked to perceptions of defeat, entrapment, hopelessness, isolation, negative social relationships, feeling inconsequential, and the presence of positive psychosis-related experiences, such as hearing derogatory voices and/or feeling persecuted [5–8]. Whilst research seeking to identify factors which exacerbate suicidal experiences in people with psychosis is growing, research examining psychological resilience to suicidal experiences is comparatively sparse [9, 10].

The concept of psychological resilience has been framed in diverse ways in the literature. In a position paper, Gooding and colleagues (2022) highlighted how some definitions of resilience have viewed it as an unchangeable personality characteristic [10]. A recent qualitative study aimed to gain service users’ insights into what resilience to suicidality meant to them to provide a more authentic conceptualisation based on the views of experts by experience (EBEs), rather than mainly relying on the perspectives of clinicians and academics [6]. Participants viewed resilience as comprising both ‘passive’ and ‘active’ responses to stressors. Passive responses were conceptualised as learning to live with suicidal experiences, whilst active responses were considered circumscribed behaviours and thought processes that people engaged in to cope with negative stressors. That does not mean to say that ‘passive’ means lacking in effort, insight, motivation or courage. Quite the contrary.

Two literature reviews have highlighted common factors that confer resilience to suicidal experiences in people with psychosis. The first review identified a range of protective factors in quantitative studies, which included family support, positive values, service engagement and hope for recovery, insight, and proactive reasons to live [11]. The second narrative review of both qualitative and quantitative studies documented ways in which perceived social support, religious and spiritual beliefs, reasons for living, and positive personal skills and attributes contributed to building resilience in people with psychosis. These attributes included being able to regulate behaviours, coping skills, self-esteem, and perceptions of control [12].

There are currently only two qualitative studies that have examined the perceptions of individuals with psychosis concerning resilience to suicidal experiences who had also experienced psychological therapy [13, 14]. In both studies, understanding resilience to suicidal experiences was not a primary aim so data about resilience was not explicitly sought. Instead, information about resilience to suicidal thoughts and behaviours was interpreted from participant responses to questions about related concepts and experiences. First, Awenat and colleagues (2017) sought participants’ views of a suicide-focused therapy, namely, Cognitive Behavioural Suicide Prevention for Psychosis (CBSPp) and reported that accessing positive memories, attention switching, and perspective taking were viewed as helpful by participants in countering suicidal thoughts [13]. Second, in an earlier study by Hyde (2001), participants were asked about their experiences of a support group, which aimed to facilitate the development of coping mechanisms [14]. Participants identified that reality testing through conversations with others that challenged unshared experiences, distraction, and realising that they were not alone were all beneficial. However, it was unclear whether these were considered helpful for addressing suicidal thoughts specifically, or mental health problems more generally.

Psychological interventions that target suicidal experiences are few, but those that have been published evidence feasibility, acceptability, and potential effectiveness in a range of populations, including those with psychosis [15–20]. However, psychological processes underpinning resilience to suicide following engagement in psychological therapy that targets suicide experiences have, thus far, not been examined. Therefore, the current study aimed to investigate the formation, development, and maintenance of psychological resilience from the perspective of individuals who had engaged with psychological therapy.

## Methods

### Design

This was a nested qualitative study based on one-to-one semi-structured qualitative interviews with participants enrolled in a two-armed randomised controlled trial (RCT) called Cognitive AppRoaches to coMbatting Suicidality (CARMS; [21]). This study is one of a series of qualitative studies that have utilised the data obtained in the CARMS project. The trial arms comprised the control group (treatment as usual [TAU]) and the treatment group (cognitive behavioural suicide prevention therapy [22] plus TAU). The study was approved by the Northwest-Greater Manchester Research Ethics Committee (17/NW/0089).

### Experts-by-experience (EBE) and Service user Engagement in Research

An EBE group (self-titled ‘the CARMers’) contributed to the development of CARMS throughout the lifecycle of the project. The CARMers had lived experience of psychosis and/or suicidality and were recruited via their involvement in previous studies with the research team. The CARMers attended frequent meetings prior to, and during, both CARMS and the current study. Meetings took place on-line during Covid-19 restrictions, and in a hybrid format at other times. The CARMers gave input and advice across many domains. Specifically: 1. they were involved in the initial development of the project through: (a) identifying potential research questions; (b) contributing to topic guide development; (c) providing feedback on study materials such as participant information sheets; and (d) participating in piloting to provide feedback and training for the interviewers; and 2. a CARMers member was specifically involved in the analysis of the current interview transcripts who met with the lead author several times to discuss their own interpretations of the data from a lived experience perspective, and to co-develop codes and subsequent themes; and 3. the CARMers reviewed the themes, subthemes, and clinical recommendations of the current study to aid the phrasing and ensure conclusions and outputs were meaningful for individuals with lived experience of severe mental health problems.

### Recruitment and sampling

Participant recruitment took place via referrals into the CARMS project [21] primarily via community and inpatient mental health services from three National Health Service (NHS) Trusts in the North-West of the United Kingdom. Eligibility for the CARMS project, and nested qualitative study, were: (i) International Classification of Mental and Behavioural Disorders version 10 (ICD-10 [23]) for a diagnosis of non-affective psychosis (F20-F29); (ii) self-reported suicidal experiences in the three months prior to recruitment; (iii) aged 18 years or over; (iv) English speaking or having sufficient fluency in English to not require an interpreter; (v) under the care of NHS mental health teams with a care-coordinator; and (vi) able to provide informed consent in accordance with the British Psychological Society’s (BPS) guidelines [24]. Participant data were eligible for inclusion in the current study if the participant indicated that they had past or current experience of engaging with psychological therapy either via CARMS, and/or independently of CARMS. Data pertaining to the number and duration of previous/current psychological therapy sessions that were not offered as part of CARMS were not recorded.

Following recruitment to the CARMS project, participants were invited to take part in separate interviews about their experiences of psychosis, suicide, and of suicide-focused therapy. Interviews were conducted at varying times following recruitment. Most participants (*n* = 32) had completed therapy prior to the interviews. Participants for the CARMS qualitative studies, including the current study, were sampled using purposive sampling to maximise variance in demographics and experiences with mental health problems [25]. The sampling was guided by a matrix consisting of: (1) scores on the Adult Suicide Ideation Questionnaire (ASIQ) which is a 25-item questionnaire comprising statements on a 7-point scale measuring suicidal ideation severity [24], (2) exposure to therapy,, (3) age, (4) gender, and (5) ethnicity.

### Procedure

Participants provided separate consent for the qualitative studies specifically. For interviews up to March 2020, participants were offered a choice over the location for face-to-face interviews i.e., at their home or in an NHS building. From March 2020, all interviews were conducted via telephone due to COVID-19 lock-down restrictions [26].

Interviews were conducted using flexible topic guides by qualitatively trained research assistants. The topic guides included questions about the pathways to suicidal experiences which probed mechanisms, escalators and de-escalators, the influence of psychotic experiences on suicidal thoughts and acts, and the perceived effects of psychological therapy on suicide and psychosis. The content of the topic guides was informed by the current literature, including work completed by the research team, the personal and professional experiences of members of the research team, and EBE insights. Open-ended questions were used to elicit participants’ views and experiences, and participants were given the opportunity to raise any additional points and reflect on their experience of the interview. The interviews also explored other aspects of psychosis and suicidal experiences in addition to psychological suicide mechanisms and the effects of therapy, such as the acceptability and feasibility of delivering suicide-focussed therapy and experiences of suicide research [27–30].

### Data analysis

Data were analysed using Reflexive Thematic Analysis (RTA) using an inductive approach at both a semantic and latent level [31]. A critical realist position was adopted because it has the ontological premise that reality exists, but that this understanding of reality is influenced by personal experiences and perspectives, including those of the participants, interviewers, and analysts [32]. An iterative coding process was used, which allowed the flexible and continuous revision of themes.

The first author (NB) led on the analysis, with support from PG and SP who also independently coded three transcripts for initial discussions and development of codes and early themes. An EBE researcher/co-author (AS) also coded with the first author/lead analyst, and after coding was completed aided theme construction.

The data analysis process was guided by Braun and Clarke’s (2021) six stages of reflexive thematic analysis. Specifically, transcripts were read and re-read many times for familiarisation (Stage 1), which aided the identification of data relevant to the research question. As part of the familiarisation process, a selection of interviews was listened to by the first author/lead analysist to facilitate a sense of closeness with the participants’ experiences, gain a greater contextual understanding of the data, to gain a sense of the paralinguistic aspects, such as tone of voice, and to reflect on their own initial reactions to the transcripts which they documented within their reflexive log. Transcripts were then initially coded within Microsoft Word, by highlighting the chosen text and providing commentary, reflections, and interpretations using comment boxes (Stage 2). Coding was limited to data that were directly related to the research question. However, as noted in some of the reflexive log entries, some data contained information that could have been interpretated as related to differing concepts. For example, in some cases it was unclear whether the participants referred to the impact of therapy on resilience to suicidal experiences specifically, or mental health and wellbeing more generally. These cases were considered alongside the rest of the transcript and the authors’ interpretation of the context to which the data referred. The EBE researcher was maximally involved in discussions of this nature. Codes from co-analysts were compared and discussed in regular data discussion sessions, with reasons for differing interpretations from professional (theoretical and clinical) and personal experiences reflected on, and discussed.

Data and initial coding were then transferred to NVivo (version 12), which was used to organise and track coding (Stage 3). Codes were repeatedly reviewed by the research team, with codes refined over time. Data were coded on both a semantic and latent level.

After all the relevant data had been coded, they were reviewed to develop patterns (i.e., candidate themes and sub-themes) and grouped together to review the viability of each candidate theme and sub-theme (Stage 4). This process involved revisiting codes several times because the understanding of the codes developed iteratively. Specifically, the coded data were combined based on shared meanings and involved, eventually, collapsing the codes to create overarching codes (i.e., candidate themes). The input of the EBE researcher/co-analyst was central to this process.

The candidate themes and subthemes were then reviewed by the full team to determine whether they provided a clear ‘story’ based on the dataset and research question. As in other stages, previous stages were returned to in the analysis and coding revised based on new novel interpretations of the data following theme generation.

Theme names were then developed, which involved searching codes within themes for nomenclature that captured the content of the theme, whilst providing a clear contribution to the research question (Stage 5). The EBE researcher/co-analyst aided the generation of theme names by a process of refinement so that they were meaningful to service users and easy to understand as a standalone phrase. Quotes from the transcripts were then selected to represent the theme, subtheme, and interpretations of the data. Multiple quotes were taken from different transcripts to ensure diversity in the representation of data provided.

**Fig. 1:**
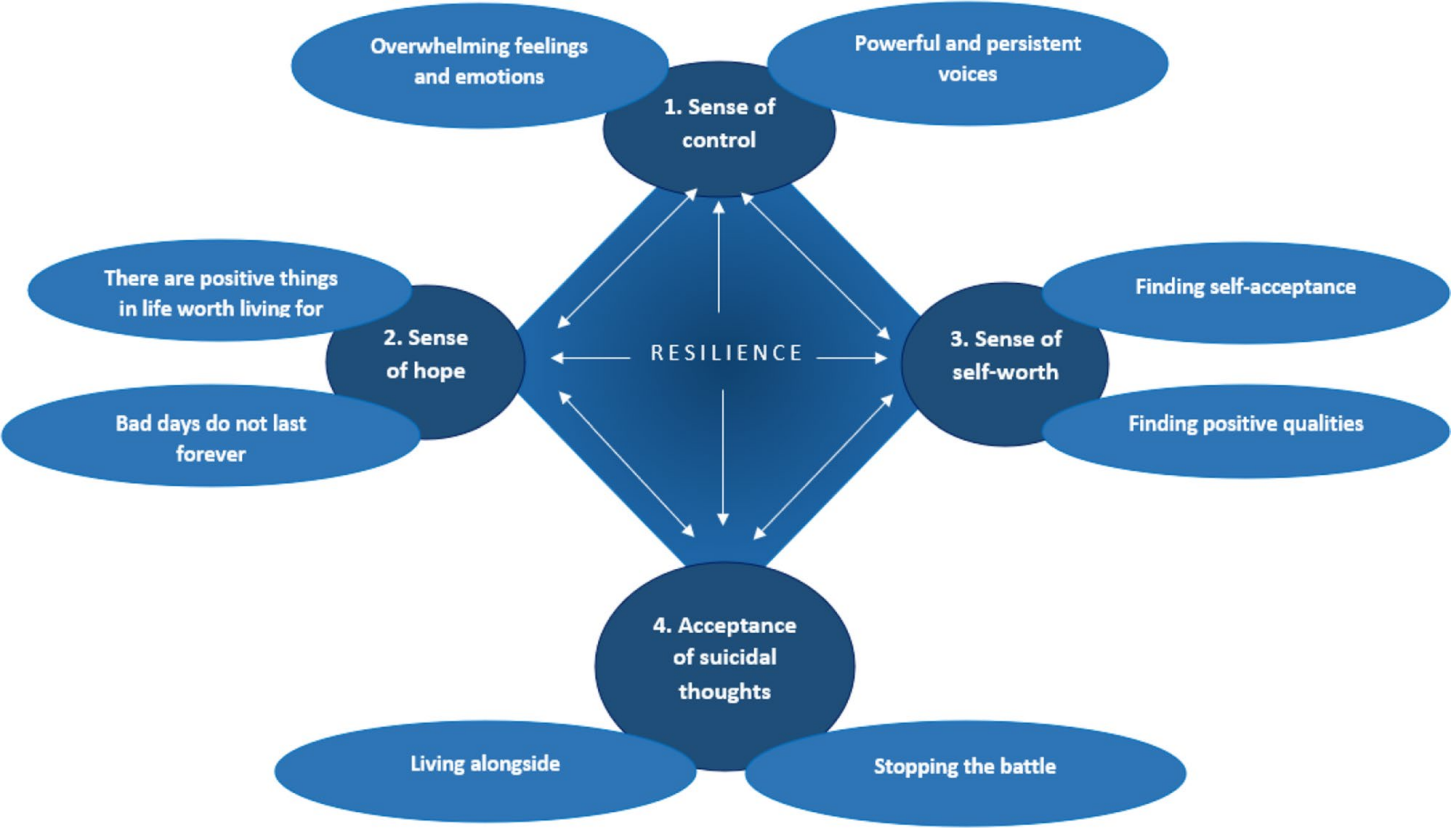
Thematic map showing the four psychological processes underpinning psychological resilience to psychotic and suicidal experiences

The final reporting stage involved the write-up of the results (Stage 6). Previous stages were returned to as the write-up progressed. This was further enhanced through co-author’s commentaries on the results write-up, with further interpretations of the data taking place when presented in a narrative. During this stage, the order of themes was established to provide a logical and meaningful narrative. A thematic map (Fig. 1) was developed to visually present the subsequent candidate themes and subthemes.

### Reflexivity and rigor

The team involved in the analysis of the transcripts comprised an EBE researcher/co-analyst, a trainee clinical psychologist, a qualified academic clinical psychologist, a health psychologist with expertise in qualitative methodology, and a non-clinical psychological scientist specialising in suicide research. Members of the research team had personal and/or professional experiences of suicidal thoughts and/or behaviours. All authors attempted to recognise and embrace the ways in which their professional and personal experiences could affect data analysis, interpretations, and recommendations [33]. Therefore, throughout the current study, the research team engaged in reflexivity though the use of reflexive notes, group discussions, keeping a record of coding and theme development, ensuring a consensus on themes, and consultation with the CARMers [34].

Whilst it is not possible to highlight all of the ways in which personal and professional experiences contributed to the analysis, a worked example is presented. Specifically, reflexivity and subjectivity are key components that distinguish reflexive thematic analysis from more traditional thematic analysis. Nevertheless, it is important that interpretations remain grounded in the data (Braun & Clarke, 2019). One example of where the first author/lead analyst had to exercise caution with regards to over-interpretation was their tendency to focus on participant’s perceptions of the value of the therapeutic relationship on the development of resilience. Indeed, it is notable that whilst many participants made positive comments about their relationship with the therapist, few commented on how this was helpful in engaging with therapeutic strategies or how this promoted resilience. An excerpt from the first author’s reflexive log reads:

I really want to capture the strong therapeutic relationship discussed by participants, but I’m finding in the vast majority of cases, that whilst participants talk about the therapeutic relationship, they do not link to how this helped in the development of resilience. I feel my draw to exploring the therapeutic relationship is related to my work as a trainee clinical psychologist, where much emphasis in clinical supervision is on the therapeutic relationship.

The first author/lead analyst was delivering clinical interventions using a Cognitive Analytic Therapy informed approach during the analysis, where the relational patterns between client and therapist are a tool for change. Therefore, they reflected on the extent to which learning this approach may have contributed to them being drawn to the therapeutic relationship within transcripts.

## Results

### Participant characteristics

There were 35 participants in the current study who were interviewed between 2017 and 2021. The duration of the interviews ranged from 22 min to 77 min, with an average duration of 44 min. The ages of participants ranged from 18 to 60 years (Mean = 36.03, SD = 12.92). Many participants were in the 25–34 age bracket (*n* = 11; 31.43%), whilst the remainder were in the 18–24 (*n* = 7; 20%), 35–44 (*n* = 6; 17.14%); 45–54 (*n* = 8; 22.86%), and 55–64 (*n* = 3, 8.57%) age brackets. Most of the sample were female (*n* = 21; 60%). A diagnosis of schizophrenia was the most common (*n* = 26; 74.29%). Other diagnoses included schizoaffective disorder (*n* = 4; 11.43%), unspecified nonorganic psychosis (*n* = 3; 8.57%), persistent delusional disorder (*n* = 1; 2.86%), and other nonorganic psychotic disorder (*n* = 1; 2.86%). The majority of the participants were unemployed/exempted from work through disability (*n* = 28; 80%), whilst the remaining participants were in paid employment/self-employed (*n* = 3; 8.57%), working voluntary (*n* = 2; 5.71%); or students (*n* = 2; 5.71%). Most of the sample were White British (*n* = 31; 88.57%). Two participants (5.71%) stated that they were Mixed Race, one participant (2.86%) identified as Black African/Caribbean/British, and one participant (5.71%) identified as Asian British. All participants had current or recent suicidal thoughts and/or behaviours (ASIQ [35]: M = 68, SD = 37.59, range = 15–130, possible range = 0–150). Many scored in the upper range, with eight participants (24.24%) scoring within the 100–150 range and 13 scoring within the 50–100 range (39.39%), with the remainder scoring within the 31–50 (*n* = 5; 15.15%) and 0–30 (*n* = 7; 21.21%) ranges. Most participants currently had psychotic experiences, namely, hallucinations, paranoia, and/or delusions.

Due to the current study being nested within the overall CARMS project, over half the participants interviewed had received, or were currently receiving, suicide-focused CBT therapy (CBSPp) (*n* = 21; 60%). Almost half of the participants reported having engaged with CBT in the qualitative interviews (*n* = 18; 51,43%). Other therapies that participants had previously received included dialectical behaviour therapy (*n* = 5; 14.29%), person-centred counselling (*n* = 3; 8.57%), cognitive analytical therapy (*n* = 2; 5.71%), and voice dialogue (*n* = 1; 2.86%). Ten participants (28.57%) indicated that they were unsure about the type of therapy they had received prior to CARMS. Finally, just under two-thirds stated that they had received multiple types of psychological therapy across their lifetime (*n* = 21; 60%). Those participants offered therapy as part of CARMS completed a mean of 17 sessions, with a range of 0 to 24.

### Overview of key findings

Four psychological processes were identified during the analysis process as being central to the development and maintenance of resilience to suicidal thoughts and behaviours in tandem with psychotic experiences: (1) a sense of control; (2) a sense of hope for the future; (3) a sense of self-worth; and (4) acceptance of suicidal thoughts (see Fig. 1).

### Theme 1: sense of control

Theme 1 had two sub-themes which were about a felt sense of control in relation to (i) Persistent and powerful voices and (ii) Overwhelming feelings and emotions. Participants described the unrelenting and controlling nature of voices. For many participants, the immense power of the voices together with perceptions of a lack of control over those voices led to suicidal thoughts and attempts. Other participants did not directly link voice hearing to suicidal experiences and, instead, viewed suicidal thoughts and behaviours as resulting from overwhelming feelings and emotions that they felt unable to control. Some individuals also perceived identifiable links between the overwhelming nature of feelings and emotions and the intensely negative manifestation of voices. Participants described how engaging in psychological therapy was instrumental to them gaining a sense of control over the voices and overwhelming emotions, which enhanced resilience to suicidal experiences. However, some participants did not necessarily report feeling in control of voices and emotions, but instead no longer felt *under* their control, and it was this feeling of no longer being hijacked by the voices and powerful accompanying emotions that aided their resilience. For individuals who had limited experience of therapy, there was the expectation that they wanted to learn how to gain control of their voices and emotions and/or be relinquished from their hold, via therapy. Feeling and perceiving an ability to *gain* control over voices or emotions that had previously seemed hijacking, is distinct from being in a psychological space where those voices and/or those emotions can no longer exert that kind of control.

### Subtheme i: persistent and powerful voices

Many participants reflected on the perceived uncontrollability of distressing, relentless, and hostile voices, which had often been present for many years. Delusional interpretations of voices as being related to external entities often exacerbated perceptions of the control and power of the voices. For example, one participant relayed that ‘Satan’ woke them up in the morning and was a presence with them throughout the day, with that presence feeling threatening, attacking, and immobilising:

*“I hear Satan speaking to me and he wakes me up in the morning and he controls me all day. He attacks me*,* you know verbally*,* or physically… He freezes me to the chair… and controls my mind”* (Participant 5).

Experiencing this kind of continual presence of one or more aggressive, antagonistic voices which did not appear to desist, reinforced a perception that the voices were omnipotent, inextinguishable, and all-pervasive. Consequently, participants often viewed suicide as the only option to escape, stop, or appease the voices *“and that’s when I start thinking of taking my life*,* because I can’t handle these voices I hear*,* just can’t handle them”* (Participant 2).

Indeed, a key aim for many participants when entering therapy was to talk about their voices and to learn different techniques that could be used to control those voices. However, a consequence of chasing control was often exhaustion, increased stress, and a consequent escalation of those voices. Furthermore, any expectation that therapy could aid explicit control or subjugation of hallucinations when that could not be realised, could potentially amplify distress. A perceived need to gain control over voices can be contrasted with the effect that gaining an understanding of how hallucinations may have arisen had on participant’s changing views of their hallucinations. By engaging with psychological therapy, participants described how they began to acquire and develop an in-depth understanding of their past and current difficulties and how psychosis and suicide-related experiences were often linked to previous traumas:

*“[My voices came from] childhood history. I’ve learnt that through therapy… it’s made me understand why I’m hearing voices and how to deal with them…”* (Participant 16).

For these participants, an increased understanding of the voices facilitated a shift in their perception of how much agency they had in directing their voices, and therefore, the effect that their voices could have on them. For example, one participant described how they learnt that their voices represented different aspects of themselves which meant that they felt able to confront the once, seemingly, unyielding, commanding disparaging, and critical voices but also embrace different protective voices. Whilst this was often described by participants as ‘gaining control’ over the voices, this perception of control seemed more reflective of an increasing sense of personal strength, self-identity, and self-understanding:

*“He’ll [voice] say*,* “Why don’t you pour that hot kettle on your hand?” Or he’ll make me paranoid to the point where I think I can’t carry on. But what I have found is my other voices… they’ll stop me. And what I’ve learnt as well*,* each voice is a represent of me. So*,* it’s me*,* but all different… I find having them more beneficial since I’ve been having therapy instead of a burden… I have the strength to tell him [the voice] off”* (Participant 11).

An increasing understanding of the voices, and subsequent feelings of agency, were often facilitated in therapy by co-creation of diagrammatic representations of past and current experiences, i.e., formulations. Participants viewed these formulations as simplifying what had originally felt complex and confusing. The consequent gains in understanding many different aspects of their past and current experiences, and the links between the past and present, seemed to stimulate and foster in participants self-compassion and hope. Moreover, challenging the reality of voice content in therapy sessions and recognising that the voices could not physically harm them, was viewed by participants as illustrating that the voices could not exert control over them. Participants no longer felt controlled by, or under the control of, their voices This resulted in perceptions of having options about voices, of having increased autonomy, and of having a sense of self which could be de-coupled from the voices, all of which acted to counter suicidal thoughts, urges and acts and build resilience because now there were alternatives. Participants continued to use their abilities to challenge their beliefs about voices in their day-to-day lives, which contributed to their growing sense of autonomy. They developed perceptions that, rather than being in control, voices were manageable, did not have the power to physically cause harm, and that they, the participants, had the skills within themselves to challenge, redirect, and/or ignore their voices:

*“It’s just knowing that I’m in control*,* even though I have voices in my head*,* it’s knowing that I’m in control*,* because nobody can hurt me only me. You know*,* the voices in my head they might be there*,* they might be saying negative things and to do bad stuff*,* but there’s only me that can actually do it to myself…they can’t actually hurt me…”* (Participant 32).

### Subtheme ii: overwhelming feelings and emotions

Participants described how their emotional difficulties could often feel overwhelming which was coupled with immense vulnerability. What seemed to come across was a kind of helplessness which emanated from these kinds of powerful emotions, perhaps, accompanied by not really knowing how, or feeling able, to communicate about them. For some individuals, a way of dealing with these sorts of emotional experiences was to try to suppress them. One participant went on to explain the pressures that he felt as a white, cis-gendered, man to behave in accord with societal expectations, one of which, as perceived by the participant, was that men should not show their emotions but, instead, keep them under control:

*“Er*,* vulnerable. Because it [talking about difficulties]*,* it taps into that … it’s like being a child again*,* essentially*,* because your emotions are some of the most powerful things that we have to deal with on a day-to-day basis. So keeping control of your emotions [is important]*,* especially as a man in this society. As a cis white male living in 2019”* (Participant 25).

One effect of therapy was described as allowing emotions to be released that participants had tried to restrain or inhibit, in some cases for many years. Some participants explained that they had used drugs, alcohol, and sometimes self-harm to try to keep emotions at bay, and under control. Whilst the potential for releasing and examining emotions that had been hidden for so long did not appear to be distressing for participants, there was, nevertheless, a detectable wariness of therapy expressed by some individuals. This wariness was due to fears of negative consequences, or indeed, the unknowns, of explicitly allowing powerful emotions to surface after prolonged periods of repression:

*“It [therapy] brought them back up after being repressed for about twenty years… I wouldn’t say struggling [with those]*,* but they’re there. And it’s like just trying to readjust. I don’t want to regress to how I readjusted because I buried all those shitty emotions under a mountain of drugs and alcohol.”* (Participant 28).

Participants relayed how their tendencies to bottle-up or withhold emotions had been reinforced because their attempts to talk and share had been met by off-putting reactions and extreme or invalidating responses from friends and family members. Such reactions by others conveyed to participants that talking about their difficult experiences and emotions was unacceptable and, thus, had to be hidden or inhibited:

*“I don’t talk to anyone about things regarding my BPD [Borderline Personality Disorder] and my delusions*,* my paranoia… I won’t talk to any of them [relatives] about that because… it causes uproars and things like that”* (Participant 22).

Due to these types of reactions, participants appeared to have kept overwhelming emotions to themselves, and felt that they had no outlet for expression or relief. It was in these situations, that suicidal thoughts, urges, and behaviours came to the forefront of their minds with some participants using self-harm behaviours to provide a release and gain a felt sense of control. Participants explained how engaging in psychological therapy empowered them to start to share difficult thoughts and feelings with their therapists. They described a sense of relief when they felt able to express their emotions as opposed to feeling compelled to control, suppress or inhibit them.

The relationship that was formed with therapists was viewed as important in helping participants to build autonomy by determining the focus and direction of therapy and contributing to them feeling empowered. When therapists were viewed as understanding, non-judgmental, and trusted, then it helped promote participants’ confidence in talking about their overwhelming experiences, feelings, and emotions which, in turn, accentuated their feelings of self-worth. Moreover, when therapists were perceived as explicitly working collaboratively with participants in discussing issues and content that they, the participant, felt were important, then this engendered participants’ feelings of ownership, control, and autonomy during therapy sessions. This also applied to practical arrangements such as when therapists offered choices over the timing, duration, and location of therapy:

*“I like the fact when he [therapist] comes in*,* he asks how long do I want it and stuff*,* and I really appreciate that… I feel like it’s*,* I’m in control of the therapy*,* it’s my therapy so I’m in control of it”* (Participant 11).

Embracing the role of a collaborative partner in these kinds of decision making during the therapy process by participants was both empowering and enabling because it allowed them to gain confidence in their abilities to navigate and live with difficult emotions and experiences outside the therapy room in their day-to-day lives. For what felt like the first time for many, participants were able to determine for themselves what would happen and when. This seemed in direct contrast to their experiences of feeling so controlled by voices and so overwhelmed by emotions that they experienced a kind of ensuing paralysis and helplessness.

### Theme 2: sense of hope

This second theme captured how participants often had an enduring and recurring set of perceptions and beliefs that nothing could help them when they faced, and had to live with, exceptionally distressing experiences, for example hallucinations which vividly re-enacted abuse. This, in turn, led participants to feel hopeless because it appeared to them that nothing could or would change. Consequently, they appeared to feel completely trapped with no reason to keep going. Suicide was seen as the only way out:

*“ It just feels like*,* just like no matter what way I turn or no matter what way I try to do anything that it’s not going to change anything”* (Participant 3).

Participants explained how psychological therapy initiated in them a recognition that changes were possible and that aspects of their lives carried a felt value that was worth living for. These changes were perceptible and tangible, and elicited a sense of hope in participants which had previously been absent. It is important to note that for some participants, they did not necessarily develop a sense of hope, but stopped feeling so utterly hopeless. An important part of these transitions away from hopelessness was the recognition that a bad day was just a moment in time. Hence, this theme comprised two sub-themes of (i) Bad days do not last forever, and (ii) There are positive things in life worth living for.

### Subtheme i: bad days do not last forever

Participants who had engaged with the suicide-focussed therapy in the CARMS project described how they learnt to recall multi-sensory positive memories in therapy which immersed them in an emotional and sensory warmth evoked by those memories, but also imparted a knowledge that they had felt ‘happiness’ in the past, and so could have those kinds of experiences again. Participants were guided by therapists in using extensive imagery techniques to aid memory recall. With this guided imagery, participants brought the memory to life vividly and interactively through all five senses. The resultant emotions in-the-moment instilled a sense of hope, and palpably so:

*“Thinking of a positive memory and going over it in my head. And imagining yourself there and thinking like*,* what was the smell and different senses…it makes me smile… And it shows that you don’t have to be in a dark place forever”* (Participant 24).

Some participants relayed how talking with therapists led to them recalling traumatic life experiences, particularly child abuse, that fed into a process of increased understanding of their current day-to-day problems and experiences. Intimate partner violence, grieving for the loss of loved ones, children being taken into care, relationship breakdown, substance use, and criminal activities were just some of the life events perceived to be impacting current situations. Whilst recalling these past traumatic experiences could be distressing, participants also described a process of reflection where they were able to recognise how far they had come in their lives despite the challenges and barriers that they had encountered. This recognition fostered participants’ beliefs that change was, and is, possible for them which elicited hope, built resilience, and countered suicidal thoughts and plans:

*“Maybe we have to look back to be grateful to where we are now…. If I look back then I’m more grateful to – that I’m much better than I used to be. So*,* that felt positive”* (Participant 33).

For some participants, the realisation that negative thoughts and feelings could improve over time and would not last forever was promoted through completing thought records introduced during therapy. The thought records were used to capture thoughts and feelings about a situation together with evidence for and against negative thoughts. Participants could see how their thoughts and feelings changed and fluctuated throughout any one day, and from one day to the next. For example, one participant used a mood rating scale of 0–10 during therapy, which helped them observe how low mood, hopelessness, and suicidal thoughts could come and go and were not static, thus making them feel less hopeless:

*“So that [thought record] was interesting to look at to go… even though the days are different… tomorrow it might be a ten*,* today it’s a three”* (Participant 22).

### Subtheme ii: there are positive things in life that are worth living for

Participants explained how therapists often encouraged them to identify and acknowledge the aspects of their day-to-day lives that were worth living for, no matter how brief or seemingly small, which could act to off-set suicidal thoughts. These *“moments of joy”* (participant 18) could replace an excessive focus on more difficult or challenging aspects of their lives. Taking time to stop and notice such moments could help participants recognise that there were experiences that were worth living for, and this, in turn, buffered suicidal thoughts:

*“He [the therapist] taught me to try and look for a moment of joy. There’s not many moments of joy when you have paranoid schizophrenia. But if you can look for a moment of joy it can be worth so much… A different thought*,* just for a second. That’s all it takes. Suicide takes a second… but if you just replace that second with a positive second”* (Participant 18).

Similarly, participants indicated that identifying and naming protective and/or preventative factors with therapists augmented the recognition that there were aspects of their lives that were worth living for, providing reasons to disengage with suicidal thoughts and an incentive to continue living:

*“When I discussed suicide with them [the therapist] they asked me questions like what’s preventing you and we went through like the reasons*,* so I listed those reasons*,* it gave me more desire*,* more ambition*,* more reason to live”* (Participant 33).

Whilst identifying moments that evoked a kind of emotional warmth and subsequent hope was described as a valued strategy by many participants, one individual relayed feeling “devastated” when the therapist suggested this as a technique. Their life seemed so hopeless to the participant that the therapist’s suggestion led to the perception by that participant that they were not being listened to:

*“It was just the way that he [the therapist] said think of three nice things and I just snapped inside… I’ve got no washing machine*,* as you can see. I’ve got no fridge freezer… I’m in trouble… So*,* you know my life’s upside down… he wasn’t listening”* (Participant 17).

When participants thought that difficult life experiences were being dismissed, then this resulted in them disengaging from therapy and no longer seeming to trust the therapist, highlighting that for them, focusing on aspects of their lives that promoted a sense of wellbeing could not nullify or eradicate the challenging experiences that they were currently facing. Therefore, for some people whose life circumstances feel so inexorably harsh at the time of therapy, rather than evoke a sense of hope, these strategies may intensify perceptions of not being understood or being invalidated, thus leading to potential disengagement and increased feelings of hopelessness.

### Theme 3: sense of self-worth

Participants articulated that feelings of low self-worth were linked to thoughts of being better off dead. Low self-worth was often exacerbated by negative voice content through accusations, insults, and the voices stating that the world would be a better place if they, the participant, were not in it:

*“They [voices] tell me to*,* that I’m not worthwhile and I should just do the world a favour and stop breathing… it makes me feel like I agree with them”* (Participant 6).

Moreover, participants’ sense of self-worth, and subsequent suicidal thoughts, were negatively impacted by self-comparisons, often connected with their beliefs that others around them were achieving more. The perceived achievements of others seemed founded on societal expectations of what ‘should’ be accomplished. As one participant explained, although they strived to meet these kinds of expectations, the effort and the actual perceived accomplishments were never enough:

*“I just used to keep punishing myself*,* it was like*,* well that’s not enough*,* look you should be doing a job*,* you should be learning how to drive*,* you should have been having a degree*,* you should be doing this*,* you should be doing that*,* and none of it felt enough”* (Participant 22).

Participants discussed how psychological therapy increased their self-acceptance and their ability to both identify and appreciate their positive qualities. This subsequently fed-into to a sense of evolving self-worth that negated punishing self-perceptions of not trying hard enough, of being inadequate, and expectations of continued, inevitable, failure. These experiences have been represented by two sub-themes of (i) Finding self-acceptance and (ii) Finding positive qualities.

### Subtheme i: finding self-acceptance

Participants recounted that one of the ways that they connected difficult past traumas with current psychotic and suicide-related experiences was through psychological therapy. The resultant increased awareness of the origins of psychosis and suicidal thoughts stimulated in participants an appreciation that their severe and enduring mental health problems were not only immensely understandable, but could also be rationalised, given the traumatic experiences that they had lived through. This subsequently led to reduced feelings of self-blame conjoint with increased self-acceptance, which in turn, promoted perceptions of self-worth that counteracted suicidal thoughts:

*“I understood things a bit more as well… I found it got easier to sort of understand how I felt and*,* you know*,* why I’d maybe felt like that… realised… it’s okay to… feel a certain way and I shouldn’t feel bad”* (Participant 27).

Gaining an understanding of past experiences and their effects on their current lives during therapy also promoted self-compassion amongst participants for behaviours and decisions that had previously elicited guilt feelings, including suicide attempts. For example, *‘forgiving’* themselves for past behaviours could enable participants to feel they could carry on living:

*“he’s [therapist] helped me to understand some of my behaviours*,* like when I took that overdose and everything… I understand why I did it. And then it makes me kind of forgive myself a little bit… I feel more confident that I can keep going”* (Participant 11).

### Subtheme ii: finding positive qualities

One of the skills developed during psychological therapy which participants cited as being important, was the ability to challenge negative thoughts about themselves and, instead of being taken-over by that negativity, to affirm their own value, and their own self-worth. During interviews, there was an underlying sense of the felt pressure that society places on individuals to live their lives in a specific way. Although this pressure is clearly not unique to people who have mental health problems, the impact of psychosis-related experiences appeared to make it harder for participants to live a life in line with perceived societal expectations. For example, some participants spoke of feeling forced to take gaps in their education; experiencing difficulties in working environments due to paranoia about colleagues; unrelenting voices affecting concentration in the work place; facing difficulties in maintaining friendships; and having problems with intimate relationships. Therefore, challenging negative self-perceptions appeared to be particularly valued by participants who viewed themselves as being unworthy due to perceived failures in their lives in the context of societal expectations and societal norms. Encouragement to recognise, and endorse, their qualities and personal successes in therapy sessions helped participants to develop a sense of “happiness” and appreciation with the person that they were, without feeling the need to put excessive demands and pressures on themselves, which had historically contributed to their suicidal thoughts:

*“I’ve got to appreciate myself as I am in a simple sense [since therapy]… keep things simple and not to think about like doing too much… I was caught between massive ambitions and just being young and happy… being young and happy and just being myself is more important than being like a guy who’s accomplished a lot in life*” (Participant 33).

Many participants indicated that low self-worth had led them to have thoughts that others would be better-off if they were not alive, and that no one would care if they died. For these participants, challenging these thoughts during therapy sessions and recognising that they did matter to others was pivotal in countering suicidal thoughts and attempts:

*“It’s like if I kill myself who’s going to miss me? Who’s it going to affect? And the more you think about that*,* actually I’ve got my mum*,* I’ve got my dad*,* I’ve got my sister*,* I’ve got the animals… All the people that I’ve made a decent impact on”* (Participant 25).

For some participants, feeling able to embrace their positive qualities was viewed as so fundamental in reducing and eradicating suicidal experiences that they set this as a therapeutic goal. For example, one participant told us how therapy encouraged them through homework to make a note of the qualities that other people had noticed and shared about them, to help reinforce their realisation and ownership of their own qualities. They noticed a change in themselves whereby they were able to start to listen to what others were communicating and, most importantly, recognise and embrace their own qualities as a consequence.

Similarly, when therapists were perceived to have genuinely appreciated and acknowledged the achievements of participants during therapy then this appeared to be seen by participants as crucial for improving their sense of self-worth. Participants recounted how it could often be challenging for them to identify and recognise their achievements themselves. Therefore, therapists noticing and naming these achievements helped participants to notice them themselves subsequently.

### Theme 4: acceptance of mental health problems and suicidal thoughts

The interviews revealed different aspects of acceptance which comprised this fourth theme. For example, acceptance could represent a kind of reconciliation about having mental health problems, specifically schizophrenia. This was sometimes accompanied by a change of perspective which involved embracing aspects of the condition:

*“It’s made me accept my schizophrenia*,* and that this is a long-term condition. But I’ve also learnt the positives about it*,* like I mentioned before. So*,* I feel like it’s changed my viewpoint on myself and what I’m going through”* (Participant 12).

Participants also spoke about two slightly different, but nevertheless inter-related, aspects of the processes underlying acceptance which were represented by two sub-themes of (i) Stopping the battle and (ii) Living alongside suicidal experiences.

### Sub-theme i. stopping the battle

Throughout the interviews, participants used terms such as “fight” and “battle” to describe how they had previously tried to manage suicidal thoughts, urges, and attempts; psychosis-related experiences; and difficulties in their day-to-day lives, prior to engaging with psychological therapy. Participants conveyed how they felt that their lives were taken up with fighting a battle. Central to feeling that each day was a fight, were participant’s specific psychotic experiences. Hearing voices that became increasingly hostile as the day progressed, or feeling almost paralysed by fear at the thought of going out because other people were watching them was more than a struggle. For many it came across as being terrifying. It should also be pointed out that the daily ‘fight’ that participants felt that they faced had numerous aspects to it. For example, many individuals struggled with finance, housing, and child care which was made worse by the additional complexities of stigmatised attitudes from others which could prohibit conversations about resources and/or seeking help.

One way of dealing with this feeling of having to constantly fight was to keep fighting. To not be kept down. However, many interviewees did feel exactly that, namely, knocked down and defeated. Moreover, when participants tried to keep fighting, they appeared to became more and more exhausted. An interesting alternative to fighting was presented in the form of acceptance. This kind of acceptance took both great insight and courage in that individuals learned to accept that they did feel low, and that things felt really ‘bad’ for them right now in many ways. What seemed crucial was to be able to ‘stay with’ those difficult moods, difficult emotions, and difficult states of mind, and to live alongside those states without necessarily trying to change them. This took effort, determination, and endurance. Implicit in this idea of ‘staying with’ difficult thoughts and emotions was a mind-set that difficult mental health problems may fluctuate and that they do not have to be all-encompassing:

*“… I’m going to be like this for a while and then I’ll be better again*,* kind of. So*,* instead of like*,* trying to fight it*,* just like*,* [short pause] stay with it and like*,* there’s no point in trying to like*,* battle it*,* just do whatever makes you like*,* happy. So*,* if like lying there*,* if I was happy lying there*,* then there’s no point in not lying there”* (Participant 29).

Learning to use distraction was an important part of being with, rather than fighting, psychotic and suicidal experiences for some participants. For example, one person described how sitting and watching television provided a redirection for negative thoughts and/or a way to halt such thoughts. Although this distraction process was viewed by some participants as a relatively passive process, turning attention from negative thoughts to something else, took substantial effort. In contrast, other participants found distraction as an isolated technique to be unhelpful. For these participants, it seemed that they invested a lot of effort into using distractions as a technique and, when these were not helpful, it produced frustration alongside a type of hopelessness, in the form of a belief that nothing works or helps despite the clear effort that had been expended.

### Ii. Living alongside suicidal experiences

Acceptance was also developed by participants through learning to live with, and live alongside, psychotic and suicidal experiences This type of acceptance-based resilience manifested as not trying to get rid of, or diminish suicidal thoughts, but letting them be, and letting them pass:

*“Well*,* I’d normally just be… lying down on the settee watching TV and then it [the suicidal thought] would just wash over me. Then I’d just have to go*,* ‘oh god here we go again’ and just let it disappear”* (Participant 26).

A different approach that helped participants establish this type of acceptance was through a cognitive diffusion technique that involved creating a visual image of the suicidal thoughts floating away:

*“[The therapist] took me through a YouTube video where I have to focus negative thoughts onto a leaf which is flowing down a stream*,* and imagine that the thoughts have been transferred”* (Participant 35).

This technique appeared to enable participants to recognise that they did not have to focus on negative thoughts, and they could let them be and allow them to pass, and that this did not result in negative repercussions. This ability provided an alternative to feeling forced to concentrate on, give attention to, and engage with, negative thoughts, including suicidal thoughts, thus reducing their impact:

*“So I just try – I try and just let it [suicidal thought] pass*,* I just let it pass… It’s not overwhelming anymore”* (Participant 33).

An interesting point arising from this last quote is that allowing suicidal thoughts to move, to pass through, also seemed to reduce feelings of being overwhelmed by such thoughts. Feeling overwhelmed, was in itself, often highly distressing.

Acceptance was not portrayed as neutralising the gravity of living with suicidal and psychotic experiences. Instead, it represented a different way of viewing those experiences and learning to be with them despite the concomitant distress. It should be noted that acceptance could feel impossible for some participants. The intensity of the voices and thoughts felt too much to be able to accept, and the urge to fight and battle was too strong. This challenges the notion that acceptance is a passive process requiring minimal effort. Rather, acceptance appeared to require immense effort and insight, together with an ability to tolerate uncertainty, particularly the uncertainty that mental health problems would, actually, fluctuate, sometimes becoming less intense, frequent, or severe.

## Discussion

This study aimed to understand psychological resilience to suicidal and psychotic experiences using qualitative methods and analyses. Perhaps the most striking observation was that participants did evidence resilience in the face of living with severe mental health problems. Other studies using qualitative [36–42], quantitative [43], and mixed methods [6, 44, 45] have also illustrated resilience processes in people with psychosis who were suicidal [12]. However, this is the first study to examine the perspectives of people with recent suicidal and psychotic mental health problems who could draw on their experiences and reflections of psychological therapy to show how resilience to suicidal and psychotic experiences can be instantiated and developed. There were four key findings of this study.

The first key finding was rather than battle or fight distressing hallucinatory or emotional experiences, learning to live alongside or finding ways to nullify them aided the development of resilience. Negative hallucinatory images and voices have been shown to be one of the most pernicious catalysts for suicidal thoughts and acts [8, 46, 47] which was echoed by our participants. Our findings illustrated how negative thoughts and perceptions accompanying psychotic experiences could change from being compelled to battle, fight, or avoid these experiences, which largely resulted in exhausted defeat and consequent escalating suicidal thoughts/acts, to finding ways of neutralising or offsetting them, or coexisting with them.

This involved two dimensions of control. The first was to actively gain control over voices and become confident in, for example, reprimanding the voices, or redirecting their focus. The second dimension of control involved not actively attempting to acquire control over voices or overwhelming emotions, but instead, to no longer feel controlled by, or hijacked by, voices and/or emotions. Not feeling controlled by an entity, is different from acquiring control over that entity. This distinction is important, because exerting control, especially over powerful feelings and emotions, may not be not possible, desirable, or indeed, beneficial to health. The desire for control may be a product of cultural beliefs and expectations that have been perpetuated, perhaps, more extensively in Western societies, which may have contributed to individuals’ beliefs, and subsequent distress, about a perceived lack of control over emotions in particular. Furthermore, any expectation that one role of therapy is to help in exerting control over emotions, for example by suppression or avoidance is misplaced, and should be countered.

The extant literature has highlighted ways of working with voices so they are no longer perceived as controlling because their origins, triggers, and amplifiers can be understood. This means that some individuals find it possible to live alongside voices, and even have a relationship with them [48–50]. Our findings extend this by emphasising the role of less combative, more accepting approaches when psychosis, suicidal experiences, and powerful emotions interact. Future related hopelessness is one of the strongest predictors of suicidal thoughts, attempts and deaths [51–53]. People living with psychosis experience very high levels of hopelessness [54–56] which are dominant in pathways to suicidal thoughts and plans [57]. The second key finding from the current study was that participants were able to discover a sense of hope in themselves. This tapped into resilience processes which underscored that distress and negative experiences, even if intense, can be time-limited and that despite negative experiences and distress seeming to permeate day-to-day existence there were still aspects of life prevailing alongside that distress which were of tangible value. Furthermore, there were perceptions that negative memories, thoughts, and emotions do not necessarily have to be all-pervasive and define the future.

From therapists’ perspectives, working with hopelessness can feel challenging [53, 58]. The choice of therapeutic techniques will vary depending on each client. Nevertheless, one technique which participants in the current study found particularly helpful and which could be easily transported outside of the therapy session was the multi-sensory elicitation of positive memories, known as Broad-Minded Affective Coping (BMAC) [59]. The BMAC has been used previously with people who have psychosis and are suicidal, albeit in more experimental settings [13, 60]. The technique has also been associated with reductions in suicidal ideation in university students [61]. Our findings illustrate the applicability of this technique in therapy settings in people accessing secondary care mental health services. Becoming immersed in positive memories served two purposes (i) to invoke in-the-moment positive emotions, and (ii) to emphasise that situations and events evoking those memories had occurred and, hence, could do so again. The theoretical origin of the BAMC was in the Broaden-and-Build Theory of positive emotions [62, 63]. There is a growing literature demonstrating (i) the cognitive-emotional adaptive significance of positive emotions [64]; (ii) links between broaden-and-build mechanisms and resilience [43, 65–70]; and (iii) the role of positive emotions in not only building hope in people who are suicidal [71], but also in undoing the effects of negative emotions [72, 73]. Hence, broaden-and-build resilience mechanisms have immense potential for therapeutic utility for both clients [74] and therapists [75]. An important point was raised that techniques such as Broaden-and-Build had the potential to seem shallow given the magnitude of psychological, societal, and medical difficulties that participants were experiencing at the time suggesting that a thorough, individual assessment and collaborative formulation of the appropriateness of this approach should be taken with clients.

Negative and self-critical thoughts, such as perceptions of being a constant failure, feeling inferior compared to others, or feeling a burden, have been found to be associated with amplified suicidal experiences [76–78]. People with psychosis have explained how these sorts of thoughts can be fuelled by hallucinations and lead to increased suicidal thoughts and behaviours [7, 37, 44, 79–81]. All of these sorts of experiences were relayed by participants in the current study. However, our third finding showed how a sense of self-worth could emerge amidst these thoughts and feelings of being of no, or of little value. We found that although self-worth was interwoven with accessing qualities in the self, in accord with work demonstrating the buffering properties of positive self-appraisals [43], it was also rooted in self-acceptance. An important component of self-acceptance was a compassionate understanding of ways in which past traumas, such a child sexual abuse, substantially contributed to mental health problems. Whilst there is a wealth of literature demonstrating associations between many different forms of child abuse and neglect, and suicidal experiences [82–84], it is vital to understand how to make this knowledge accessible to people in ways that allow them to integrate it with their own experiences so that it makes a perceptible and enduring difference to their self-understanding.

The significance of acceptance was expanded by participants and constituted the fourth novel finding of the current study. It contrasts with ideas that are often cited in resilience work of “bouncing back”, “being strong”, and “pulling through” following difficult experiences [85]. It suggests that, for some people, resilience is a multilayered process of learning to live with thoughts, emotions and experiences. Exploring acceptance across multiple domains is important, especially because previous qualitative research has demonstrated how some therapists have difficulties with understanding and accepting client’s suicidal experiences, potentially resulting in a ‘knee-jerk’ reaction towards risk management, rather than acceptance that suicidal thoughts, urges, compulsions and plans, can and do fluctuate [86].

The development of theories and models of psychological resilience to suicidal experiences has been somewhat overlooked. Promising resilience frameworks have included two-dimensional buffering models (e.g., perceptions of problem solving weaken the relationship between social isolation and suicidal thoughts), personal recovery models, and the maintenance model [9, 10]. Aspects of our findings fit with each of these models. However, they seem to illustrate most prominently the advantages of the maintenance model which was developed in the context of living with chronic physical pain [87]. A key feature of this model is the ability to find and embrace personal meaning and values whilst compassionately accepting, and co-existing with mental and/or physical health problems. This model also seems to offer the most potential for developing therapeutic interventions with a focus on harnessing and augmenting resilience to suicide in ways that embrace different forms of acceptance [10].

### Strengths and limitations

There were three main limitations of the current study. First, most of the participants were White British and actively recruited into a suicide-intervention project [21]. Resilience to suicide in marginalised individuals, and in those who are underserved might emphasise different cultural, interpersonal, and cognitive-emotional dimensions of resilience to psychotic and suicidal experiences, which may have been omitted in this study. For example, people from individualistic or collectivistic cultures may have differing stressors that lead to suicidal experiences, such as increased stigma towards mental health problems, or, conversely, protective factors that aid resilience, such as a greater sense of belonging. Additionally, responses to psychological formulations and psychoeducation highlighting the reasons for psychosis-related experiences may meld less-well with beliefs of non-Western cultures. Co-produced psychological therapies for people with psychosis from ethnic minority backgrounds are in development [88–91]. Whilst this does not mitigate the sampling limitations of the current study, it demonstrates the positive steps in the field towards developing co-produced and evidence based culturally sensitive psychological interventions for people who experience psychosis.

Second, resilience is conceptually complex with little consensus in the literature regarding defining characteristics [10, 12]. Four members of the research team had extensive prior-research projects and publications in the area of resilience to suicide meaning that these pre-formed perspectives may have weighted the interpretation of the data. That said, the research team were diverse in their professional and personal experiences of suicidality and mental health problems including psychological resilience to those problems, the analysis was inductive in nature, and the final analysis was discussed with the EBE group, which off-sets this limitation, at least to an extent.

Third, participants were not directly asked during the qualitative interviews about resilience nor what resilience meant to them personally. Nevertheless, the topic guide did probe pathways to, and mechanisms underlying, suicidality, and explored continua of escalators and de-escalators of suicidality. It also probed reactions to therapy including what helped to counter psychotic and suicidal experiences. Furthermore, that resilience was not a direct focus of interview questions is a potential strength because the term could provoke strong reactions in people with respect to the availability of mental health resources which may have over-shadowed interviewees’ responses and reduced their breadth of impact.

Two further strengths of the current study should also be highlighted. First, the EBE Group (CARMers) fed-into different stages of the work, including the development of the research question. Furthermore, an EBE researcher was closely involved in the analysis process, who had also provided consultation throughout the lifecycle of the project, for example recruitment procedures and topic guide development. This type of investigator triangulation aided data interpretation processes in collaboration with the overall research team and aided the development of themes relevant to the participants interviewed [92–94]. Second, purposive sampling meant that participants had diverse suicidal experiences, ranging from fleeting thoughts of life not being worth living to multiple suicide attempts. Moreover, suicidal experiences had all been within the past three-months of recruitment to the overall trial. Being able to capture the effects of acute and fluctuating suicidal experiences is not often possible in research studies and lends reassurance that the findings translate into real-life experiences encountered in therapy.

### Clinical implications

There are four key clinical implications. First, people with severe mental health problems, specifically psychosis and suicidality, can and do develop and maintain psychological resilience and this should be explicitly acknowledged and expanded [9, 12]. Second, different ways of compassionately accepting mental health problems including the complexities of psychotic and suicidal experiences have the potential to be insightful and beneficial for both therapists and clients. Third, and relatedly, in addition to suggesting active strategies which ‘boost’ resilience, ‘neutralise distress’ or ‘promote strength’, approaches which explore living alongside distressing experiences, thoughts and emotions may be more appealing to some clients. Fourth, exploring with clients ways that positive emotions can be used to offset negative focussed attention, relive positive events, and counter in-the-moment stressors, is to be encouraged [59].

## Conclusions

This study found evidence for four key psychological processes that contribute to resilience to psychotic and suicidal experiences. Identification of these processes is important because they emphasise clients’ perceptions about the principles of change that promote resilience. This can subsequently guide future psychological intervention development, with greater integration of strategies across different psychotherapeutic approaches. Future resilience research should (i) examine the effects on resilience of different therapeutic modalities and settings, aside from cognitive therapies and individual one-to-one therapy, and (ii) seek to determine which therapeutic techniques optimally promote resilience to suicidal and psychotic experiences.

## Data Availability

The raw datasets generated and analysed during the current study are not publicly available because participants did not provide consent.

## Declarations

### Disclaimers

The views expressed in this publication are those of the author(s) and not necessarily those of the MRC, NIHR, or the Department of Health and Social Care. The CARMS trial is hosted by Greater Manchester Mental Health NHS Foundation Trust, UK. The Sponsor is the University of Manchester, UK.
